# Errata

**Published:** 2006-02

Azziz-Baumgartner et al. noticed two errors in “Case–Control Study of an Acute Aflatoxicosis Outbreak—Kenya?” [ Environ Health Perspect 113:1779–1783]. The units in Figure 2 and Table 2 should be nanograms per milligram instead of micrograms per milligram. The errors were introduced when new figures and tables were generated during the final revision of the paper. The authors apologize for these errors.

In the article by Feist et al. [ Environ Health Perspect 113:1675–1682], the units were incorrect in several figures and tables: “Lipid (μg/g)” should be “μg/g lipid” in Tables 1 and 2 and in the *y*-axes of Figures 2 and 3A–C. Also, on the *y*-axes in Figure 5A–D, “dL” should be “mL.” *EHP* regrets these errors.

The photograph on page A29 of the January 2006 NIEHS News section should have been credited to Jennifer Gorenstein/UTMDACC COEP. The photographs on page A30 should have been credited to Tom Van Biersel/Louisiana Geological Survey (left) and Bryan Parras/UTMB (right). Additionally, Parras’s photograph depicts residents of Pointe-aux-Chenes, not LaRose, and includes no COEP staff.

In the Beyond the Bench article in this same section, “COEPs Contribute to Hurricane Relief” [ Environ Health Perspect 114:A30–A31 (2006)], Peter Thorne was incorrectly identified as director of the University of Iowa COEP; he is in fact director of the University of Iowa Environmental Health Sciences Research Center as well as head of the NIEHS Working Group on Mold, Microbial Agents, and Respiratory Diseases. It was the latter group that “collected air and surface samples from water-damaged homes in New Orleans” as our article stated. Finally, the aid teams that traveled throughout Louisiana included members from the UTMDACC COEP as well as the UTMB COEP.

*EHP* regrets the errors.

In the January Focus article “In Katrina’s Wake” [ Environ Health Perspect 114:A32–A39 (2005)], Hurricane Katrina was identified as a Category 4 storm, reflecting statements from the National Hurricane Center as of press time. The National Hurricane Center has since reported that Katrina was actually a Category 3 storm at the time of landfall.

